# Treatment adherence, persistence, and effectiveness of fixed dose combination versus free combination therapy of rosuvastatin–ezetimibe as a lipid-lowering therapy

**DOI:** 10.3389/fcvm.2025.1461416

**Published:** 2025-05-16

**Authors:** Mihail Samnaliev, Irfan Khan, Praveen Potukuchi, Kelly Lee, Genevieve Garon, Charlie Nicholls

**Affiliations:** ^1^ Department of RWE and HEOR Practice, Axtria, Boston, MA, United States; ^2^ Department of General Medicines Real World Evidence, Sanofi, Bridgewater, NJ, United States; ^3^ Department of General Medicines and Market Access, Sanofi, Reading, United Kingdom; ^4^ Global Medical Department of General Medicines, Sanofi, Toronto, ON, Canada

**Keywords:** fixed-dose combination, free-combination treatment, single-pill combination, persistence, adherence, major adverse cardiovascular events, rosuvastatin, ezetimibe

## Abstract

**Background:**

Guidelines for dyslipidemia management recommend adding ezetimibe for patients with dyslipidemia inadequately controlled with statin monotherapy. A fixed-dose combination (FDC) of statin and ezetimibe may improve persistence and adherence and hence reduce LDL-C further compared to free-combination treatment (FCT). The primary aim was to compare persistence/adherence with FDC versus FCT of rosuvastatin and ezetimibe (R/E); the secondary aim was to assess the impact of treatment adherence and persistence to LDL-C percentage reduction from baseline. An exploratory analysis assessed the impact of treatment adherence and persistence to incidence of major adverse cardiovascular events (MACEs). A subgroup analysis of patients on FDC of rosuvastatin 10 mg and ezetimibe 10 mg was also conducted.

**Methods:**

A retrospective analysis was performed using the THIN® database from Belgium and France in individuals (aged ≥18 years who received R/E as FDC or FCT between January 01, 2017, and November 30, 2022). Persistence (time from landmark date to discontinuation, with the latter defined as >45 days gap between prescription fills) and adherence (having a proportion of days covered ≥80%) were defined. Subsequent analyses adopted propensity score matching or weighting, followed by Cox and logistic regression models.

**Results:**

A total of 15,643 treatment episodes (FDC: 11,300; FCT: 4,343) were selected. FDC R/E was associated with greater persistence (HR: 0.54, 95% CI: 0.51–0.58) and higher odds of adherence (OR: 3.00, 95% CI: 2.70–3.30) than FCT R/E. Based on the regression analysis results, patients who were persistent to treatment had a 10% higher reduction in LDL-C values from baseline than those non persistent. Similarly, patients who were adherent had 9.6% higher reduction in LDL-C levels from baseline than those not adherent. No significant difference was observed in association between persistence/adherence and MACEs. A consistent trend was also observed in the subgroup analysis.

**Conclusions:**

In conclusion, FDC of R/E use was associated with higher treatment persistence and adherence than FCT of R/E. Patients persistent/adherent to treatment had greater LDL-C reductions than those who discontinued or did not follow treatment schedule. The limited number of MACEs suggests a cautious interpretation of exploratory MACE findings.

## 1 Introduction

Cardiovascular disease (CVD) continues to be the leading cause of mortality and morbidity worldwide, accounting for 3.81 million deaths overall in 2021 (1). In 2021, CVD accounted for 0.695 million deaths in the US, 1.7 million deaths in the European Union, and 4 million deaths in China (2–5). Evidence showed that lowering low-density lipoprotein cholesterol (LDL-C) levels is crucial for reducing the risk of atherosclerotic cardiovascular disease (ASCVD) and major adverse cardiovascular events (MACEs) (6, 7). The European Society of Cardiology (ESC)/European Atherosclerosis Society (EAS) guidelines for the management of dyslipidemia recommend using a lipid-lowering therapy (LLT) to achieve target LDL-C levels of <1.4 mmol/L (<55 mg/dl) and <1.8 mmol/L (<70 mg/dl) for patients at very high and high cardiovascular risk, respectively (7). Although the landscape of LLTs has evolved, many patients with high cardiovascular risk (CV) fail to achieve guideline-recommended LDL-C goals.

Statins are recommended for preventing mortality and morbidity associated with CVD, due to their lipid-lowering and plaque-stabilizing effects (8). However, despite the widespread use of statins, a significant proportion of patients remain at risk of CV events. Recent data from the European SANTORINI (9) and DA VINCI (10) registries and the US GOULD national registry (11) have shown that even with an optimized statin therapy, a substantial proportion of patients do not achieve guideline-recommended LDL-C levels, suggesting an unmet need for LLT optimization. Further intensification of LLTs, such as the addition of ezetimibe to statins, is recommended for patients on statin monotherapy who still fail to reach the guideline-recommended LDL-C goal (7, 12, 13).

Poor adherence is a major challenge in controlling LDL-C levels due to the high number of patients failing to take prescribed medications. Treatment simplification, such as reduction in the number of tablets to be taken daily, has been an effective approach to improving persistence and adherence to treatment, such as the management of hypertension (14, 15). However, such evidence of fixed-dose combination (FDC) vs. free combination treatment (FCT) of statin and ezetimibe in dyslipidemia management is limited with mixed results. While several studies showed higher adherence to FDC compared to FCT of rosuvastatin and ezetimibe (R/E) (16–18), the study by Bartlett et al. showed limited benefit of FDC over FCT (19).

Previous studies, such as those by Rea et al. (16) and Katzmann et al. (17), have assessed the effectiveness of all-statin and ezetimibe combinations and shown that the single-pill combination of statin and ezetimibe exhibited better adherence and a larger reduction in LDL-C compared to two-pill combination of these drugs (16, 17). In this study, we compared the effectiveness of FDC vs. FCT of ezetimibe and rosuvastatin on adherence, persistence, LDL-C reduction, and incidence of CV events in a population representing a real-world clinical practice setting.

## 2 Methods

### 2.1 Study design and study population

**Figure 1:**
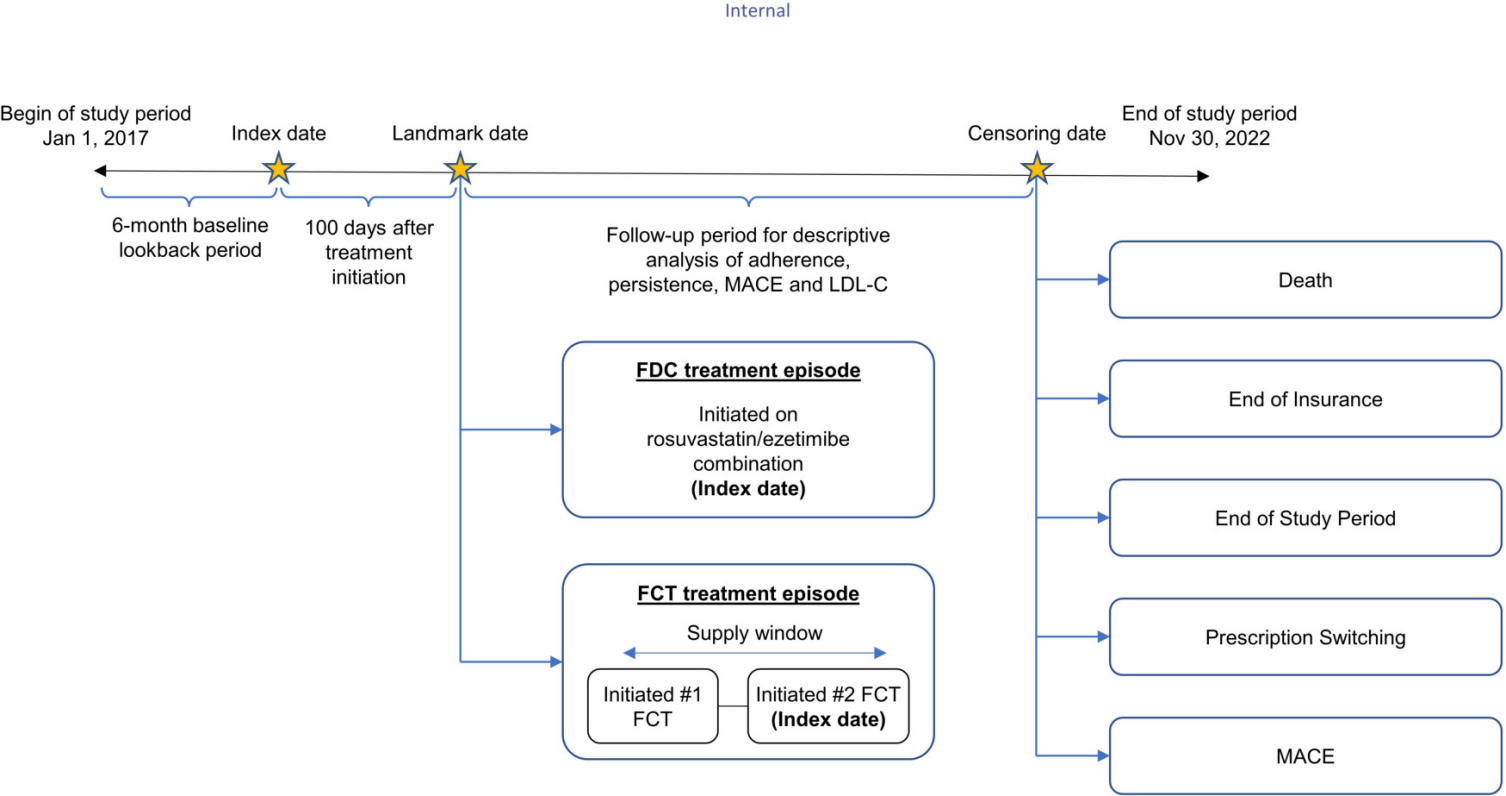
Study design. FCT, free-combination treatment; FDC, fixed-dose combination; LDL-C, low-density lipoprotein cholesterol; MACE, major adverse coronary event.

This retrospective study was conducted using primary care practice data from Belgium and France available in The Health Improvement Network (THIN) European database, accessed through the Cegedim Health Data portal. Patients over the age of 18 prescribed with FDC or FCT of rosuvastatin/ezetimibe between January 01, 2017 and November 30, 2022 were included in the study. Patients were followed until the occurrence of a MACE or censoring, including death, loss of follow-up, treatment switching, and end of the study (November 30, 2022), whichever occurred first (Figure 1).

The index date for the FDC R/E cohort was defined as the date of receipt of first prescription. For the FCT R/E cohort, if there was no other statin treatment between receiving ezetimibe and rosuvastatin, the date of receiving the second drug was considered as the index date for that episode. If there was another statin treatment between receiving ezetimibe and rosuvastatin, then the patient should subsequently have an uninterrupted combination of ezetimibe and rosuvastatin. In this case, the first dose of the uninterrupted combination was considered as the index date of that episode. The unit of analysis was an FDC/FCT treatment episode, which was classified as a period in which there was no change in the treatment and no gap in treatment exceeding 45 days. Patients were excluded if they had received proprotein convertase subtilisin/kexin type 9 inhibitor (PCSK9i) treatment in the baseline period or had missing or erroneous FCT/FDC prescriptions (i.e., prescriptions with treatment duration greater than 365 days). Treatment episodes of FDC and FCT in the same patient within 6 months of each episode were also excluded from the analysis. A conservative approach was adopted by excluding treatment episodes with a duration of <100 days to ensure treatment not just one-off prescription.

As this analysis represented the use of anonymized health data from secondary database, ethics approval was not required.

### 2.2 Study outcomes

The primary analysis compared patients' adherence and persistence to FDC R/E vs. FCT R/E. Persistence, based on discontinuation, was assessed from the landmark date, defined as 100 days post-index date, until 365 days after the landmark date, or until censored (i.e., patients with treatment episode duration less than 100 days were excluded from the analysis). Treatment was considered discontinued if there was no prescription refill within 45 days. For FCT, discontinuation of either medication was considered as discontinuation of the regimen. Persistence included the days of supply of the last prescription of the respective treatment episode. Proportion of days covered (PDC) was used to measure patients' adherence to treatment and calculated by total days covered by treatment (FDC/FCT) divided by time from the landmark date to the first year or censored date. Patients were considered adherent to treatment when PDC ≥ 80%.

The secondary analysis assessed the impact of treatment adherence and persistence to LDL-C change. The change in LDL-C was estimated as the difference between the LDL-C measured during a 15-month follow-up and LDL-C measured at index or the most recent prior value (within 90 days prior to index). The associations between persistence/adherence and LDL-C change were evaluated in the pooled population receiving FDC R/E or FCT R/E.

A further analysis assessed the impact of treatment adherence and persistence to MACE incidence. MACEs were defined as the following events identified in the database: myocardial infarction (MI), stroke, and hospitalized unstable angina (UA). Any death recorded within 2 weeks of a MACE was assumed attributed to cardiovascular events (20). The associations between persistence/adherence and MACE incidence were evaluated in the pooled population receiving FDC or FCT. Exploratory analyses comparing the treatment of FDC R/E vs. FCT R/E on the incidence of MACEs was also conducted.

A subgroup analysis of patients taking an FDC or FCT of rosuvastatin 10 mg and ezetimibe 10 mg (R10/E10) was also conducted using the same methodology as that for the overall analysis.

### 2.3 Statistical analysis

A 1:1 propensity score (PS) matching was implemented via a logistic regression model with the following variables measured at baseline: age, sex, body mass index (BMI), year of index, LDL-C group (divided into three categories: <70 mg/dl, 70–130 mg/dl, and >130 mg/dl), high-density lipoprotein cholesterol, systolic blood pressure (SBP), diastolic blood pressure (DBP; using four categories for SBP and DBP based on quartiles), MI, stroke, UA, hypertension, chronic obstructive pulmonary disease, heart failure, and diabetes mellitus type 1 and type 2. Besides PS matching, exact matching on low-moderate (rosuvastatin 5 mg/ezetimibe 10 mg and rosuvastatin 10 mg/ezetimibe 10 mg) vs. high (rosuvastatin 20 mg/ezetimibe 10 mg, and rosuvastatin 40 mg/ezetimibe 10 mg) intensity medication dosage at index was also implemented. Standardized mean differences were assessed across baseline covariates pre- and post-matching, and a cutoff of 0.1 was selected to represent imbalance.

The primary analyses were based on PS-matched treatment episodes. A Kaplan–Meier analysis was used to describe the time to treatment discontinuation for the FDC and FCT groups. Patients were censored at death, switching treatment (i.e., either between FCT and FDC or to another statin, or statin combination, or other LLT treatment, including PCSK9i), experience of MACE, or loss of follow-up. A multivariable Cox regression model was used to analyze treatment discontinuation associated with FDC and FCT as reference on the matched sample and further adjusted for age, sex, baseline BMI, index year, baseline laboratory test results, baseline MACE occurrence, and baseline comorbidities. A multivariable logistic regression model was used to estimate the association between the use of FDC vs. FCT and treatment adherence (PDC ≥ 80%) on the matched sample and further adjusting the above-mentioned variables. Sensitivity analyses were further conducted to assess the robustness of the result, including assessing unmatched cohorts, inverse probability of treatment weighting (IPTW), and univariate analysis.

The secondary analysis only considered treatment episodes for which both baseline and follow-up LDL-C measurements (LDL-C cohort) were available. Univariable and multivariable ordinary least squares (OLS) regression were used to evaluate the association between persistence/adherence and percentage change in LDL-C in the pooled FDC and FCT samples. Covariates considered in the multivariable OLS regression model were LDL-C baseline value, duration between the measurement of baseline and follow-up LDL-C (days from the landmark index date to the first measurement of LDL-C), comorbidities, dose group (high/low), and a country indicator (France vs. Belgium). The temporality between the predictors (persistence/adherence) and the outcomes (change in LDL-C) was observed by measuring persistence/adherence in the period prior to the measurement of LDL-C during the follow-up (i.e., patients could not alter their adherence/persistence in response to the LDL-C measurement, which would cause reverse causality). A sensitivity analysis was also performed by considering the different set of covariates in the statistical model.

For the third analysis, the temporal associations between treatment persistence/adherence and MACE incidence (time to the first MACE after the landmark date) were conducted using all FDC and FCT episodes, irrespective of the treatment received (i.e., pooled FDC and FCT analysis) and restricting to patients without any MACE prior to their landmark date to minimize the bias attributed to baseline risk (MACE cohort). For treatment persistence, the patient's persistence with FDC or FCT prior to the current time point was used as a predictor for MACE incidence. This approach accounted for the dynamic nature of persistence over time. A longitudinal logistic regression model was used to handle the repeated measures of persistence over time for each episode, accounting for within-patient correlation along with time-varying covariates (21). For treatment adherence, a time-to-event analysis using a Cox proportional hazards model was selected because of its suitability for evaluating the impact of adherence during fixed periods of time until MACE incidence. The different analysis approach selected for treatment persistence and adherence was to capture both the time-varying nature of medication use and the impact of the initial prescription period. Models were adjusted for age, sex, baseline BMI, index year, baseline laboratory test results, and baseline comorbidities (excluding baseline MI, stroke, and congestive heart failure). A sensitivity analysis by different cutoff points of treatment persistence and adherence was performed to assess the uncertainty of the result.

An additional exploratory analysis was conducted to estimate the association between the use of FDC (compared to FCT) and MACE. This analysis included only patients without a prior MACE during their baseline period prior to the landmark date to avoid potential confounding driven by the likelihood that patients with a history of MACE, who were expected to be at higher risk of a subsequent MACE, may also be more persistent to treatment. This exploratory analysis followed an intent-to-treat approach, in which patients were classified into an FDC vs. FCT group depending on whichever was initiated first. Cox models with IPTW (inverse probability of treatment weights and stabilized weights) and further adjustment for potential confounders were used to assess the robustness of the estimates. Using IPTW allowed the inclusion of all patients in the analyses compared to propensity score matching (PSM), where matches could not be found for many patients.

Missing data were not considered to be associated with the predictors and outcomes and hence assumed missing at random.

## 3 Results

Of the 43,085 patients identified in the database, 15,643 distinct episodes (FDC: 11,300; FCT: 4,343) met the selection criteria and were included in the analysis (Supplementary Figure S1). In total, 3,281 matched pairs were included in the final cohort. Baseline characteristics of the original and matched cohort patients are summarized in Table 1. For the R10/E10 subgroup, a total of 8,333 treatment episodes (FDC: 6,735; FCT: 1,598) were identified and 1,244 matched pairs were included in the analysis.

**Table 1:** Baseline characteristics of overall and matched cohort of FDC and FCT.

Baseline characteristic	Before matching			After propensity score matching		
	FDC R/E *N* = 11,300^a^	FCT R/E *N* = 4,343	SMD^b^	FDC R/E *N* = 3,281	FCT R/E *N* = 3,281	SMD
Age, mean (SD)	66 (12)	68 (13)	0.107	68 (12)	68 (13)	0.094
Female, *n* (%)	4,443 (39)	1,729 (40)	0.010	1,438 (44)	1,389 (42)	0.030
Medication usage anytime prior to the index date, *n* (%)						
Prior use of FCT (R/E)	258 (2)	742 (17)	0.5169	86 (3)	538 (16)	0.4832
Prior use of FCT (other statin + ez)	603 (5)	372 (9)	0.1272	172 (5)	322 (10)	0.1739
Prior use of FDC	429 (4)	17 (0)	0.2415	89 (3)	17 (1)	0.178
Prior medication pattern, *n* (%)						
Statin only	6,792 (60)	947 (22)	0.8466	1,969 (60)	814 (25)	0.7633
Statin high intensity^c^	1,087 (10)	90 (2)	0.3259	267 (8)	74 (2)	0.2673
Statin low-moderate intensity^d^	5,705 (50)	857 (20)	0.6805	1,702 (52)	740 (23)	0.6365
Statin + ezetimibe	831 (7)	1,087 (25)	0.4948	253 (8)	833 (25)	0.4905
Ezetimibe only	429 (4)	213 (5)	0.0533	135 (4)	187 (6)	0.0721
Medication dosage at index date, *n* (%)						
Low-moderate dose (≤10 mg) rosuvastatin	6,763 (60)	3,215 (74)		2,551 (78)	2,551 (78)	
R5/E10	28 (0)	1,617 (37)	1.0763	9 (0)	1,343 (41)	1.1629
R10/E10	6,735 (60)	1,598 (37)	0.4692	2,542 (77)	1,208 (37)	0.9013
High dose (20–40 mg) rosuvastatin	4,537 (40)	1,128 (26)		730 (22)	730 (22)	
R20/E10	4,239 (38)	1,040 (24)	0.297	686 (21)	673 (21)	0.0098
R40/E10	298 (3)	88 (2)	0.0405	44 (1)	57 (2)	0.0322
Comorbidities (baseline period), *n* (%)						
All hypertension	2,616 (23)	772 (18)	0.1332	726 (22)	625 (19)	0.0755
Essential (primary) hypertension	2,591 (23)	760 (17)	0.1352	722 (22)	617 (19)	0.0787
Chronic obstructive pulmonary disease	257 (2)	82 (2)	0.0271	83 (3)	58 (2)	0.0526
Heart failure	101 (1)	34 (1)	0.0122	36 (1)	29 (1)	0.0215
Diabetes mellitus type 1	129 (1)	40 (1)	0.0218	50 (2)	32 (1)	0.0494
Diabetes mellitus type 2	1,072 (9)	280 (6)	0.1125	314 (10)	227 (7)	0.0965
Hypercholesterolemia	1,967 (17)	631 (15)	0.0789	601 (18)	478 (15)	0.1013
Myocardial infarction	434 (4)	145 (3)	0.027	131 (4)	106 (35)	0.0408

^a^ *N* refers to treatment episodes.

^b^ SMD ≥ 0.1 can be considered as a sign of imbalance.

^c^ Atorvastatin 40–80 mg; rosuvastatin 20–40 mg; simvastatin 80 mg (no use of simvastatin 80 mg was observed) (32).

^d^ Atorvastatin 10–20 mg; rosuvastatin 5–10 mg; simvastatin 20–40 mg (moderate); simvastatin 10 mg (low) (33).

E, ezetimibe; FCT, free-combination treatment; FDC, fixed-dose combination; R, rosuvastatin; SD, standard deviation; SMD, standardized mean difference.

For medication use anytime prior to the index date, about 60% in the FDC cohort and 22% in the FCT cohort had taken statin only. More patients in the FCT cohort had taken statin and ezetimibe than those in the FDC cohort (25% vs. 7%, respectively). For medications prescribed at the index date, low-moderate intensity dose combinations (R5/E10 and R10/E10) were more frequently prescribed than high-dose combinations (R20–40 mg/E10 mg) in both FDC (60%) and FCT (74%) cohorts (Table 1).

### 3.1 Treatment persistence and adherence

**Figure 2:**
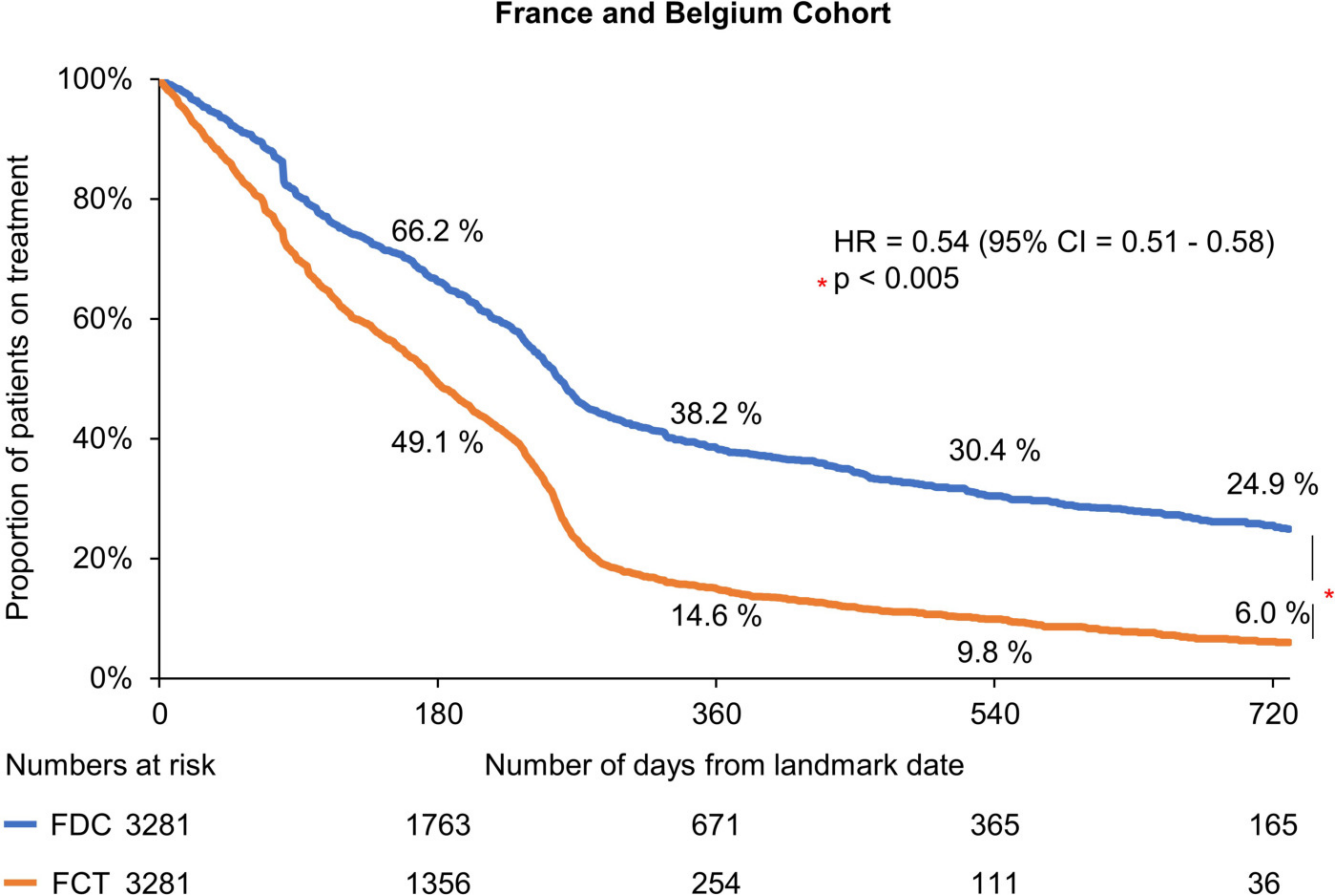
Kaplan–meier estimates of treatment discontinuation associated with FDC R/E and FCT R/E. CI, confidence interval; FCT, free-combination treatment; FDC, fixed-dose combination; HR, hazard ratio; R/E, rosuvastatin and ezetimibe.

The median time to treatment discontinuation for the matched cohort was higher for patients with FDC R/E than those with FCT R/E (200 vs. 142 days: unadjusted analysis). The 365-day persistence rate from the index date was 48% and 27% in the FDC R/E and FCT R/E cohorts, respectively. In the multivariable Cox regression analysis, patients in the FDC R/E cohort were less likely to discontinue treatment as compared with those in the FCT R/E cohort (HR = 0.54, 95% CI: 0.51–0.58) (Figure 2).

The subgroup R10/E10 analysis results showed a similar trend; the median time to discontinuation was higher with FDC than that with FCT (216 vs. 149 days: unadjusted analysis). The 365-day persistence rate from the index date was 58% and 24% in the FDC and FCT cohorts, respectively. The multivariable Cox regression model also demonstrated that patients on FDC were associated with higher treatment persistence than those on FCT, with an HR of 0.51 (95% CI: 0.47–0.56; *p* < 0.005).

In the PS matched analyses, more patients in the FDC R/E cohort were adherent to treatment than those in the FCT R/E cohort (52.70% vs. 27.60%). In the adjusted logistic model, patients in the FDC R/E cohort were more likely to be adherent compared with those in the FCT R/E cohort [odds ratio (OR): 3.00, 95% CI: 2.70–3.30; *p* < 0.001]. The subgroup R10/E10 analysis also demonstrated a consistent trend where the FDC R/E cohort had more patients adherent to treatment than the FCT R/E cohort (52.2% vs. 26.8%), and FDC R/E was associated with higher odds of adherent to treatment than FCT R/E (OR: 3.06, 95% CI: 2.57–3.64; *p* < 0.005).

### 3.2 Association between persistence/adherence and LDL-C percentage change

A total of 1,468 treatment episodes (FDC: 930; FCT: 538) that had both a baseline and a follow-up (up to 15 months) LDL-C measurement were identified. The average duration between baseline and follow-up was 203.8 days [standard deviation (SD): 73 days], and the median duration was 188 days (range: 141–260 days).

The mean (SD) of LDL-C during the baseline and follow-up period was 113 mg/dl (±44) and 76 mg/dl (±37), respectively, equivalent to a change of 36 mg/dl (±44). About half (52.8%) of the patients achieved the LDL-C goal <70 mg/dl.

In the multivariable OLS regression model, patients persistent to treatment between the landmark date and the time of the first LDL-C measurement experienced a greater percentage change from baseline in LDL-C levels (mean percentage change: −10.11%, 95% CI: −14.48 to −5.74; *p* < 0.001) than those not persistent. Similarly, patients adherent to treatment had a significantly higher percentage change from baseline in LDL-C levels (mean percentage change: −9.62%, 95% CI: −13.40 to −5.80; *p* < 0.001) than those not adherent. Patients adherent to treatment had a mean LDL-C of 9.1 mg/dl (−12.7 to −5.5) lower than that of non-adherent patients.

The subgroup R10/E10 analysis showed a consistent trend as the overall cohort. Patients persistent to treatment had a greater LDL-C percentage change from baseline (−9.9%, 95% CI: −15.7 to −4.1; *p* < 0.005) than those not persistent. Similarly, patients adherent to treatment had a greater percentage change from baseline in LDL-C levels (−11.2%, 95% CI: −16.1 to −6.3; *p* < 0.005) than those not adherent. Patients adherent to treatment had a mean LDL-C 10.4 mg/dl (−15.2 to −5.4) lower than non-adherent patients.

### 3.3 Association between persistence/adherence and MACE incidence

Overall, 166 patients had experienced MACEs during the follow-up period (from the landmark date to the first occurrence of MACE or censoring event), which included 126 MIs, 25 strokes, 18 hospitalizations due to UA, and 1 CV-related death. No statistically significant difference was observed in MACE incidence between persistent and non-persistent patients at 90 days after the landmark date (OR: 1.21, 95% CI: 0.84–1.75; *p* = 0.311). Similarly, the association between treatment adherence status and MACE incidence was also not statistically significant (HR: 0.90, 95% CI: 0.60–1.36; *p* = 0.620).

The subgroup R10/E10 analysis results were consistent with the main analysis; no significant association was observed between persistence/adherence and MACE incidence.

### 3.4 Exploratory analysis

The association between FDC (vs. FCT) and MACE was based on 14,288 unique patients (without prior MACEs before the landmark date), of which 10,406 patients were treated with FDC R/E and 3,882 were treated with FCT R/E. The average follow-up period of the cohort was 211 days and generated 166 outcomes, including 126 MIs, 25 strokes, 18 events of hospitalized UA, and 1 CV-related death. The percentage of patients without MACEs initially, at 90 days (99.6% vs. 99.7%) and 180 days (99.5% vs. 99.3%) was similar across the FDC R/E and FCT R/E cohorts. However, the FDC cohort had a higher percentage of patients without experiencing MACEs over a longer period, including at 365 days (99.0% vs. 98.4% for FDC vs. FCT) and at 2 years (98.7% vs. 97.5% for FDC vs. FCT).

In the multivariable Cox PH model, patients in the FDC R/E cohort had a lower risk of experiencing MACEs than those in the FCT R/E cohort (HR: 0.60, 95% CI: 0.41–0.88; *p* = 0.010).

The subgroup R10/E10 analysis results also showed that patients on FDC were associated with a 42% lower risk of MACEs than those on FCT (HR: 0.58, 95% CI: 0.35–0.96; *p* = 0.030).

## 4 Discussion

The findings of this retrospective analysis suggest that treatment with FDC R/E was associated with higher persistence and adherence as compared with FCT R/E. Further, in the pooled analysis of patients receiving either FDC or FCT of R/E, being persistent/adherent to treatment was associated with a greater reduction in LDL-C levels from baseline, as compared to not being persistent/adherent. While no association between persistence/adherence and MACE incidence was observed, in the exploratory analysis, patients on FDC R/E had a lower risk of MACEs than those on FCT R/E.

In the current study, 15,643 treatment episodes were identified in 43,085 patients. Although the study did not determine the proportion of eligible patients treated with combination of R/E, in previous studies, the proportion of patients who received statin-ezetimibe FDC were reported to be 3% compared with 94.2% for all statin prescriptions (22). The finding of better medication compliance for patients on FDC R/E than those on FCT R/E is aligned with the study conducted by Rea et al., assessing a difference in treatment adherence between FDC and FCT of statin and ezetimibe using national insurance data from the Lombardy region in Italy. The analysis showed better treatment adherence in patients prescribed FDC than FCT (OR:1.87, 95% CI: 1.75–1.99) (16). A similar observation was made in the management of hypertension, where Bramlage et al. reported lower discontinuation rates associated with FDC than FCT of ramipril/amlodipine (HR: 0.64, 95% CI: 0.58–0.73) and candesartan/amlodipine (HR: 0.82, 95% CI: 0.80–0.84) (23). Consistent findings were also observed in a systematic literature review and meta-analysis by Kengne et al. comparing the impact of FDC and FCT on treatment adherence in patients with hypertension and dyslipidemia (24). The current analysis further demonstrated that treatment persistence and adherence were associated with a greater percentage change from baseline in LDL-C levels, which is consistent with the existing literature demonstrating a positive relationship between treatment persistence/adherence and clinical outcomes (25). The evidence of better treatment persistence and adherence in patients prescribed FDC than FCT, which led to better treatment effectiveness despite the same formulation of drugs, highlights the value of FDC in the management of chronic diseases.

The lack of association between treatment persistence/adherence and MACE incidence in the current analysis was inconsistent with the existing literature (16, 26–29), most likely due to the limited number of MACEs in the database, attributed to insufficient length of follow-up. Rea et al. reported that the risk of CV-related mortality and hospitalization decreased by 55% (95% CI: 20–75) in patients with high adherence (>75%) compared to those with low adherence (<25%) to statin and ezetimibe, which was based on accumulated 9,430 person-years of observations and 208 outcomes (52 CV-related deaths and 156 CV-related hospitalizations) with an average 2.2 years of follow-up period per person (16). A recent observational study of adults with a newly initiated LLT including statins and ezetimibe for primary prevention of atherosclerotic CVD in Sweden also demonstrated better adherence and persistence to LLTs was associated with a lower risk of MACEs (HR: 0.90, 95% CI: 0.85–0.95), based on a cohort of 36,283 patients with a median follow-up of 2 years and 1,034 recorded MACEs, defined as MI, ischemic stroke, and all-cause mortality (28).

While the exploratory analysis results of the current study showed a significant improvement in MACE incidence in patients on FDC R/E than those on FCT R/E, the results should be interpreted with caution due to the limitation of the data, including the limited number of events and the short follow-up period. Nevertheless, the trend was consistent with the existing literature assessing the impact of a single-pill vs. multiple-pill, fixed-dose combination on clinical outcomes. Verma *et al*. conducted a population-based retrospective cohort study in Canada evaluating the impact of FDC and FCT of antihypertensive drugs on composite endpoints, including death or hospitalization for acute MI, heart failure, or stroke. The analysis showed that FDC recipients experienced less events compared to FCT recipients (HR: 0.89, 95% CI: 0.81–0.97), based on a matched cohort of 6,675 and a median follow-up time of 1,826 days (30). A similar finding was observed in a retrospective claims database study conducted in Taiwan, which reported a significant reduction in MACEs (HR: 0.85, 95% CI: 0.74–0.97) associated with FDC vs. FCT of renin–angiotensin system and thiazide diuretic in hypertensive patients (31).

The current study had several strengths. The database used in the current analysis is based on a population enrolled in primary care reflecting real-life medical practice, which allowed the study to assess the impact of patients' behavioral changes attributed to FDC and FCT. Additionally, the dataset is the longitudinal follow-up, which allowed capturing and evaluating differences in clinical benefits. Unlike the existing literature, adopting a conservative approach by only considering patients with more than 100 days of prescription to ensure the initiation of the treatment further increased the robustness of the results. Several sensitivity analyses and subgroup analyses were also performed, and the trends were consistent with the base-case analysis, further strengthening the robustness of the analysis.

However, the study also had several limitations. There was a significant amount of missing data for LDL-C measurement; less than 10% of patients had both baseline and follow-up data, which could potentially reduce the power of the analysis. Also, the dataset was only able to capture the prescription refill without knowing whether patients were taking medication, which could bias the results. The relatively short follow-up period is another shortfall, which posed challenges in capturing the long-term clinical outcomes for the FDC and FCT cohorts. In the current analysis, while different statistical methods were utilized to minimize the difference at baseline between the FDC and FCT cohorts, including PS matching approach and multivariate regression model, unobserved or latent variables not controlled in the process could still lead to a biased result. For example, in the matched cohort of FDC and FCT, low (5 mg) and moderate (10 mg) rosuvastatin were unbalanced despite the PS matching process, which could impact the analysis for difference in clinical outcomes, such as LDL-C reduction and MACE incidence. Furthermore, while some variables in the regression model might be collinear such as hypertension and SBP categories, this issue can be mitigated by the fact that many hypertensive individuals on anti-hypertensive medications may achieve SBP control, supporting the rationale of these measures providing independent pieces of information (i.e., not perfect collinearity). Incorrect and missing recording of diagnosis and previous ASCVD events in the dataset were another potential constraints and could bias the analysis results due to an unbalanced baseline CV risk between the cohorts. Lastly, as the cause of death was not recorded in the dataset, a conservative approach was adopted in which CV-related deaths considered in MACE were assumed to occur within two weeks of MI, stroke, and hospitalized UA. This assumption could potentially underestimate CV-related deaths and bias the analysis results.

In summary, the findings from this study are in alignment with previous research evidence and suggest that treatment with single-pill FDC of R/E increases treatment persistence and adherence, resulting in a greater reduction of LDL-C levels compared with a free combination of two pills. Given the limited number of MACEs in the database, this study did not observe a statistically significant association between increased persistence/adherence and MACEs. While a trend of lower MACE incidence was noted in patients treated with FDC R/E compared to FCT R/E, the results should be interpreted with caution due to the limitation of the database.

## 5 Conclusions

This real-world retrospective study suggests that patients treated with rosuvastatin/ezetimibe FDC are more persistent and adherent than those treated with FCT, which is associated with greater reductions in LDL-C levels. Large, long-term studies are recommended to validate the clinical implications of FDC on MACE outcomes.

## Data Availability

The datasets presented in this article are not readily available because the analysis was funded by Sanofi, but access to study data was limited to Axtria and Axtria statisticians completed all the analyses. Data are available from Cegedim through a commercial subscription agreement and are not publicly available. No additional data are available from the authors. Requests to access the datasets should be directed to Elodie Minnaert (Elodie.MINNAERT@cegedim.com).
