# Supplementary figures and images for: Treatment adherence, persistence, and effectiveness of fixed dose combination versus free combination therapy of rosuvastatin–ezetimibe as a lipid-lowering therapy
Source: Front Cardiovasc Med. 2025 May 16;12:1461416. doi: 10.3389/fcvm.2025.1461416 (PMC12123881; doi:10.3389/fcvm.2025.1461416)

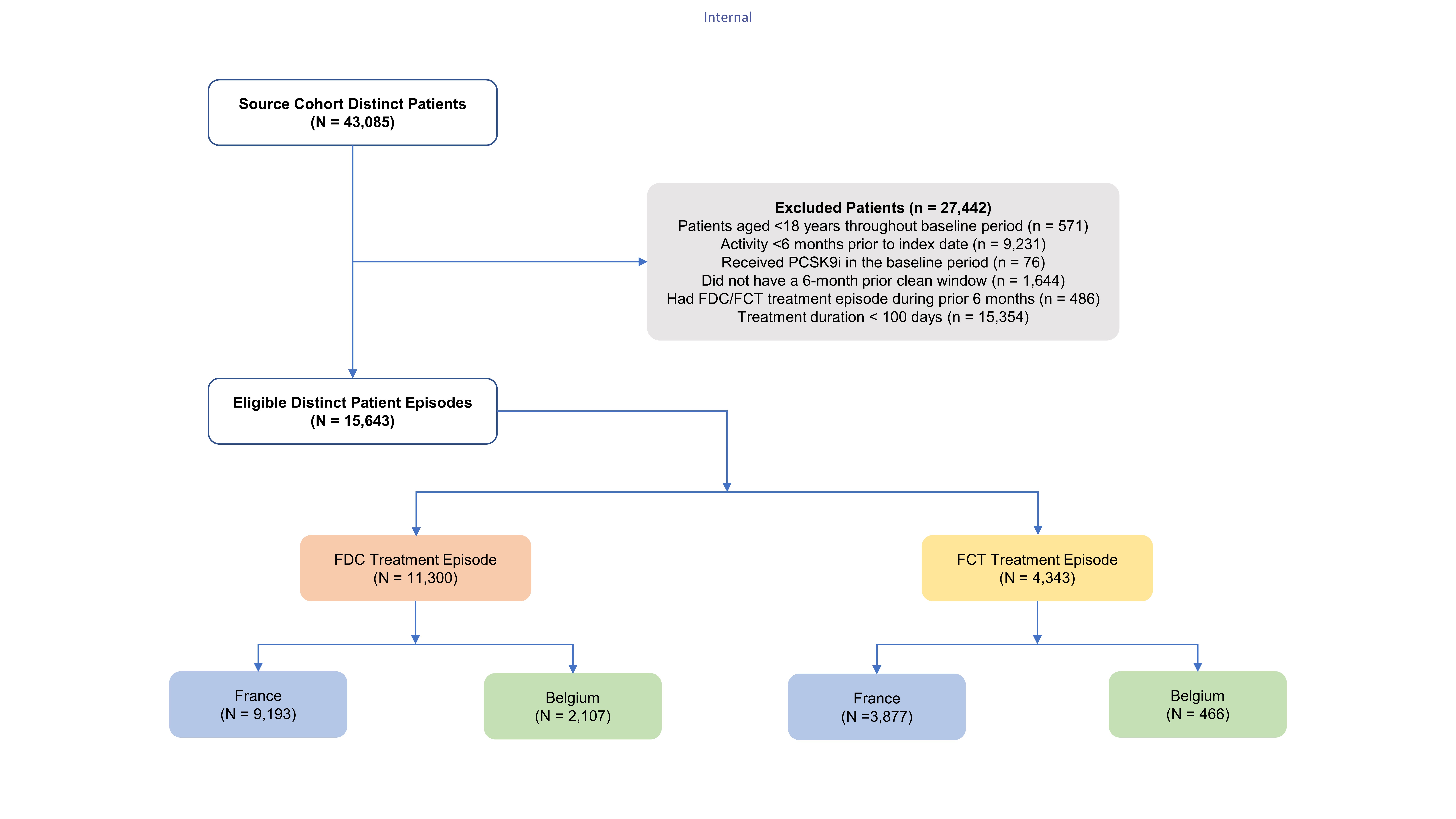

Supplement: Supplementary file 1 [file Image1.jpeg]

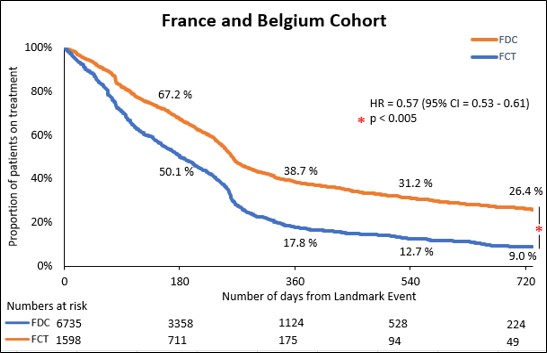

Supplement: Supplementary file 2 [file Image2.jpeg]
